# Clinical validation of Node-RADS for nodal staging in colon cancer: a retrospective study of 360 patients

**DOI:** 10.1186/s13244-026-02441-y

**Published:** 2026-11-02

**Authors:** Guo Wu, Jianning Xiao, Danfeng Wang, Yulian Xie, Kailun Lin, Han Li, Honghui Lian, Yukai Chen, Daiying Lin, Zhuangyong Xu

**Affiliations:** Department of Radiology, Shantou Central Hospital, No. 114 Waima Road, 515031 Shantou, The People’s Republic of China

**Keywords:** Colon cancer, Lymph nodes, Contrast-enhanced computed tomography, Node Reporting and Data System (Node-RADS)

## Abstract

**Objective:**

This study aimed to assess the effectiveness of the Node Reporting and Data System version 1.0 (Node-RADS 1.0) for preoperative evaluation of lymph node metastasis (LNM) in colon cancer and to benchmark its diagnostic performance against conventional CT-based size criteria.

**Materials and methods:**

This retrospective study enrolled patients who underwent radical resection for colon cancer at Shantou Central Hospital between January 2015 and January 2019. All patients received radical resection followed by histopathological evaluation of regional lymph nodes. Preoperative computed tomography (CT) images were independently reviewed by two experienced radiologists using the Node-RADS scoring system. Inter-observer agreement was assessed, and diagnostic performance was evaluated using receiver operating characteristic (ROC) analysis.

**Results:**

A total of 360 patients were included. Lymph node positivity was associated with several CT features, including nodal size, internal heterogeneity, necrosis, margin characteristics, fatty hilum status, and the Node-RADS score (all *p* < 0.05). The Node-RADS score demonstrated the highest diagnostic performance (area under the curve (AUC) = 0.835, 95% CI: 0.792–0.871) with an optimal cutoff of ≥3, good inter-observer reliability (intraclass correlation coefficient (ICC) = 0.962), and higher discriminative ability than the conventional 10-mm size criterion (AUC 0.546; *p* < 0.05).

**Conclusion:**

In this large retrospective cohort, contrast-enhanced CT–based Node-RADS demonstrated reliable diagnostic performance for preoperative assessment of LNM in colon cancer and outperformed the conventional size-based criterion. The good inter-observer agreement further supports the reproducibility and clinical feasibility of Node-RADS in routine clinical practice.

**Key Points:**

***Question*** Can CT-based Node-RADS overcome the low sensitivity and underestimation of nodal metastasis associated with the conventional 10-mm short-axis criterion in preoperative colon cancer staging?

***Findings*** In 360 patients, Node-RADS at ≥3 achieved an AUC of 0.835, 96.6% sensitivity, and good interobserver agreement, outperforming the 10-mm criterion with fewer missed metastases.

***Critical relevance statement*** In 360 patients, Node-RADS at ≥3 achieved an AUC of 0.835, 96.6% sensitivity, and good interobserver agreement, outperforming the 10-mm criterion with fewer missed metastases

**Graphical Abstract:**

**Figure:**
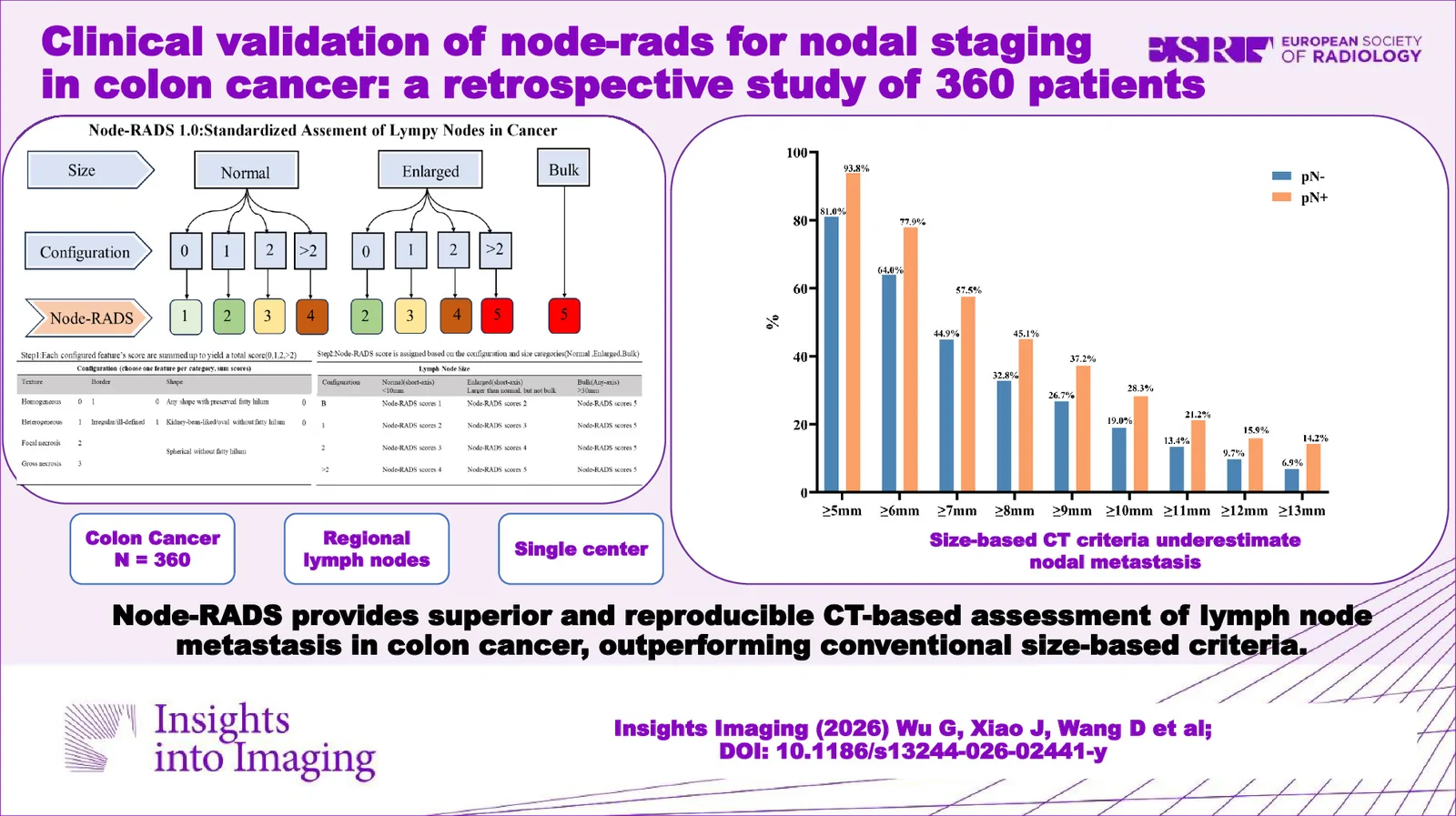

## Introduction

Colorectal cancer is a leading global malignancy, ranking third in incidence and second in cancer-related mortality worldwide [1]. Among all colorectal cancer cases, colon cancer accounts for two-thirds [1, 2]. Accurate preoperative evaluation of lymph node status is essential for TNM staging in colon cancer and is important for prognostic stratification, risk stratification, and prediction of postoperative outcomes [3, 4]. Growing evidence indicates that tumor-draining lymph nodes play an active role in antitumor immunity, while inaccurate nodal staging may lead to overtreatment and increased risk of postoperative complications [5–7].

The diagnostic performance of available imaging techniques for preoperative nodal staging in colon cancer remains suboptimal. MRI demonstrates only moderate sensitivity (61.5%) and specificity (70.3%), while positron emission tomography/computed tomography (PET/CT), despite relatively high specificity (87.9%), exhibits low sensitivity (55.1%) [8]. Although contrast-enhanced computed tomography (CT) is recommended by the NCCN guidelines for nodal staging in colon cancer [9], conventional size-based assessment using a 10-mm short-axis cutoff achieves a sensitivity of only approximately 47%, resulting in frequent underestimation of nodal involvement [10]. In the absence of standardized evaluation criteria, the overall diagnostic accuracy of CT nodal metastasis in colorectal cancer ranges from 59% to 68% [11].

In 2021, Elsholtz et al [12] proposed the first version of the Node Reporting and Data System (Node-RADS v1.0), a five-category Likert-based framework designed to standardize imaging assessment of lymph node involvement. The system enables structured reporting by estimating the probability of nodal metastasis using predefined morphological criteria, including nodal size, margin characteristics, internal heterogeneity, and shape. To date, the clinical applicability of the Node-RADS system has been demonstrated across diverse oncologic contexts, encompassing multiple organ systems, anatomical regions, and tumor types, including head and neck [13], lung [14], breast [15], esophageal [16], gastric [17], renal [18], cervical [19], rectal [20], and bladder cancers [21]. In colon cancer, Maggialetti et al [22] demonstrated that the Node-RADS score can facilitate preoperative risk stratification of lymph node metastasis (LNM). Using postoperative histopathological findings as the reference standard, they observed that the vast majority of lymph nodes categorized as Node-RADS I (scores 1–2) were non-metastatic (94.7%), whereas metastatic involvement was identified in 80.0% of Node-RADS II (score 3) nodes and 94.7% of Node-RADS III (scores 4–5) nodes, revealing a strong stepwise association between higher Node-RADS categories and pathological nodal positivity (*p* < 0.0001). Conversely, Leonhardi et al [23] reported only modest diagnostic performance of CT-based Node-RADS for nodal staging in colon cancer, with an area under the curve (AUC) of 0.68. When a cutoff score of 2 was applied, sensitivity and specificity reached 62% and 71%, respectively, while inter-observer agreement for the overall Node-RADS score remained limited (*κ* = 0.35). Given the inconsistent findings across these studies and their relatively small sample sizes (67 and 108 patients, respectively), the clinical value of Node-RADS for lymph node assessment in colon cancer has yet to be definitively established. Accordingly, the present study was conducted in a substantially larger cohort to systematically assess the diagnostic performance of CT-based Node-RADS for locoregional lymph node stratification in colon cancer and to directly compare its efficacy with the conventional 10-mm short-axis diameter criterion.

## Materials and methods

### Study design

This retrospective study was approved by the Ethics Committee of Shantou Central Hospital (Approval No. 2024-076), and the requirement for informed consent was waived. Patients who underwent radical colectomy for colon cancer at Shantou Central Hospital between January 2015 and January 2019 were consecutively enrolled.

**Fig. 1:**
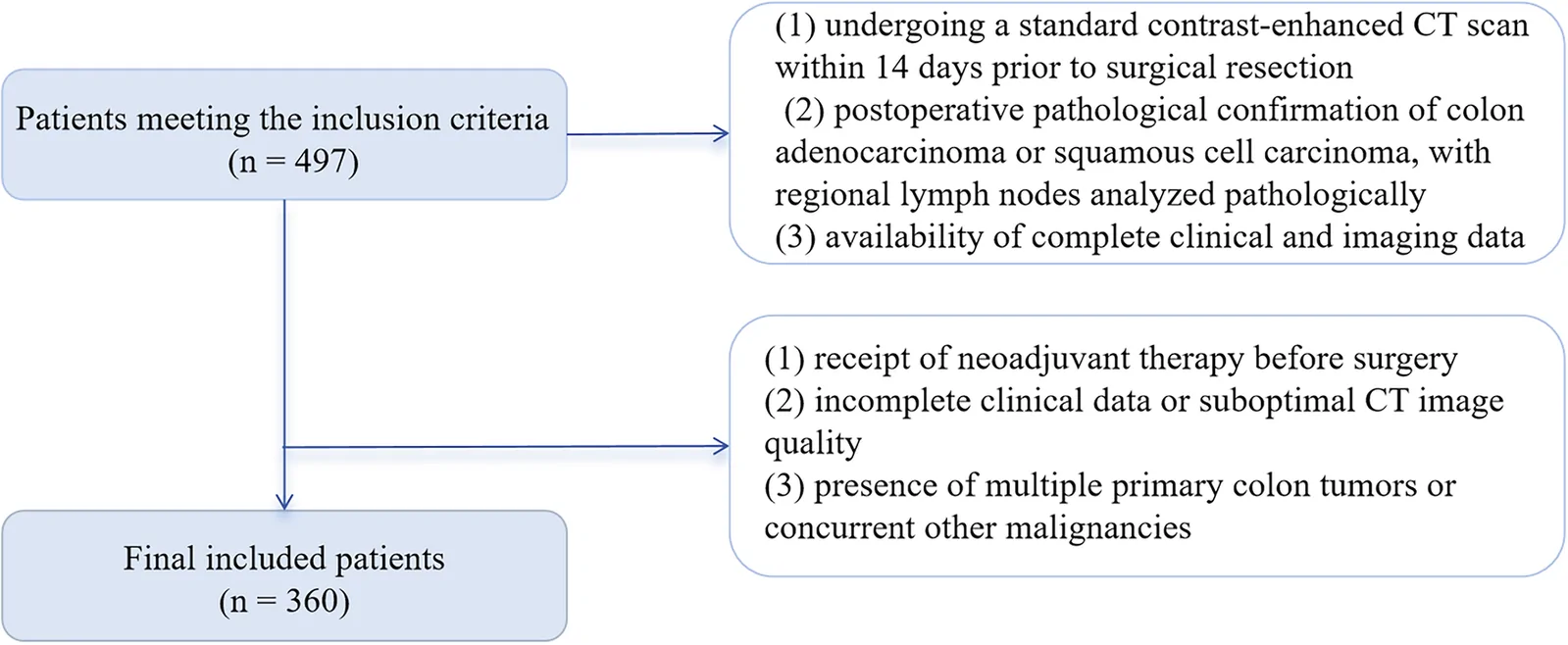
Inclusion and exclusion criteria flowchart

The inclusion criteria were: (1) availability of a standard contrast-enhanced CT examination performed within 14 days prior to surgery; (2) postoperative pathological confirmation of colon cancer with histological evaluation of regional lymph nodes; and (3) complete clinical and imaging data. Exclusion criteria included: (1) receipt of neoadjuvant therapy before surgery; (2) incomplete clinical information or inadequate CT image quality; and (3) presence of multiple primary colon tumors or concomitant malignancies (Fig. 1).

Clinical and pathological variables were retrieved from the electronic medical record and pathology databases, including age, sex, primary tumor location, histological grade, pathological T stage, lymph node metastasis status, perineural invasion, lymphovascular invasion, and distant metastasis.

### CT image acquisition

CT examinations were performed using multidetector scanners (Somatom Definition Flash 128, Siemens; LightSpeed VCT 64-slice, GE Healthcare), covering the region from the diaphragmatic dome to the symphysis pubis. After intravenous administration of a nonionic iodinated contrast agent (iopromide, 300 mg I/mL) at a dose of 1–2 mL/kg and an injection rate of 2.5–3 mL/s, arterial- and portal venous–phase images were acquired at 25 s and 70 s, respectively. Scanning parameters included a tube voltage of 120 kV, tube current of 100–200 mA adjusted for body weight, slice thickness and reconstruction interval of 1 mm, gantry rotation time of 0.5 s, a field of view of 300–400 mm, and a matrix size of 512 × 512.

### CT image assessment

Portal venous–phase CT images acquired before surgery were obtained from the Picture Archiving and Communication System (PACS). The primary tumor location was first identified, followed by systematic evaluation of the adjacent mesocolon and lymph nodes within the regional drainage basin. In this study, regional lymph nodes were defined according to the AJCC TNM classification (Version 9) as those located within the mesocolon along the colon and its feeding vessels, including pericolic, intermediate, and central lymph nodes. Among all visible lymph nodes, the short- and long-axis diameters were measured on axial images or multiplanar reconstructions that displayed the largest nodal cross-sectional area to assess morphological characteristics. Node-RADS scores were assigned to all evaluable lymph nodes, and the highest-scoring node was selected as the target. In cases where multiple lymph nodes shared the same highest score, one target lymph node was selected according to the following hierarchical rules: (1) preference for the lymph node with a larger short-axis diameter; (2) preference for lymph nodes exhibiting heterogeneous features (e.g., necrosis or indistinct borders); and (3) preference for lymph nodes with a spherical rather than elliptical shape. Morphological features assessed included internal heterogeneity, focal or gross necrosis, border definition (well-defined vs. indistinct), presence or absence of a fatty hilum, and spiculated or lobulated margins. The long-axis diameter and the corresponding short-to-long axis ratio were also documented. Lymph node assessments were independently conducted by two abdominal radiologists, each with over 9 years of experience, who were blinded to the pathological outcomes. Any discrepancies between the two readers regarding morphological features or Node-RADS scores were resolved through joint review and discussion to reach a final consensus.

### Histopathological analysis

Following radical surgical resection, specimens were processed according to standard institutional pathological protocols, primarily involving thorough manual palpation and visual inspection by experienced pathologists. Specialized fat-clearing techniques were not utilized in this study. Consistent with the American Joint Committee on Cancer (AJCC) TNM staging system, Version 9, retrieval of at least 12 lymph nodes is recommended to ensure accurate nodal staging. Across our study cohort, 5226 lymph nodes were harvested, yielding an average of 14.5 nodes per patient. Of these, 341 metastatic lymph nodes were pathologically confirmed.

### Node-RADS scoring protocol

**Fig. 2:**
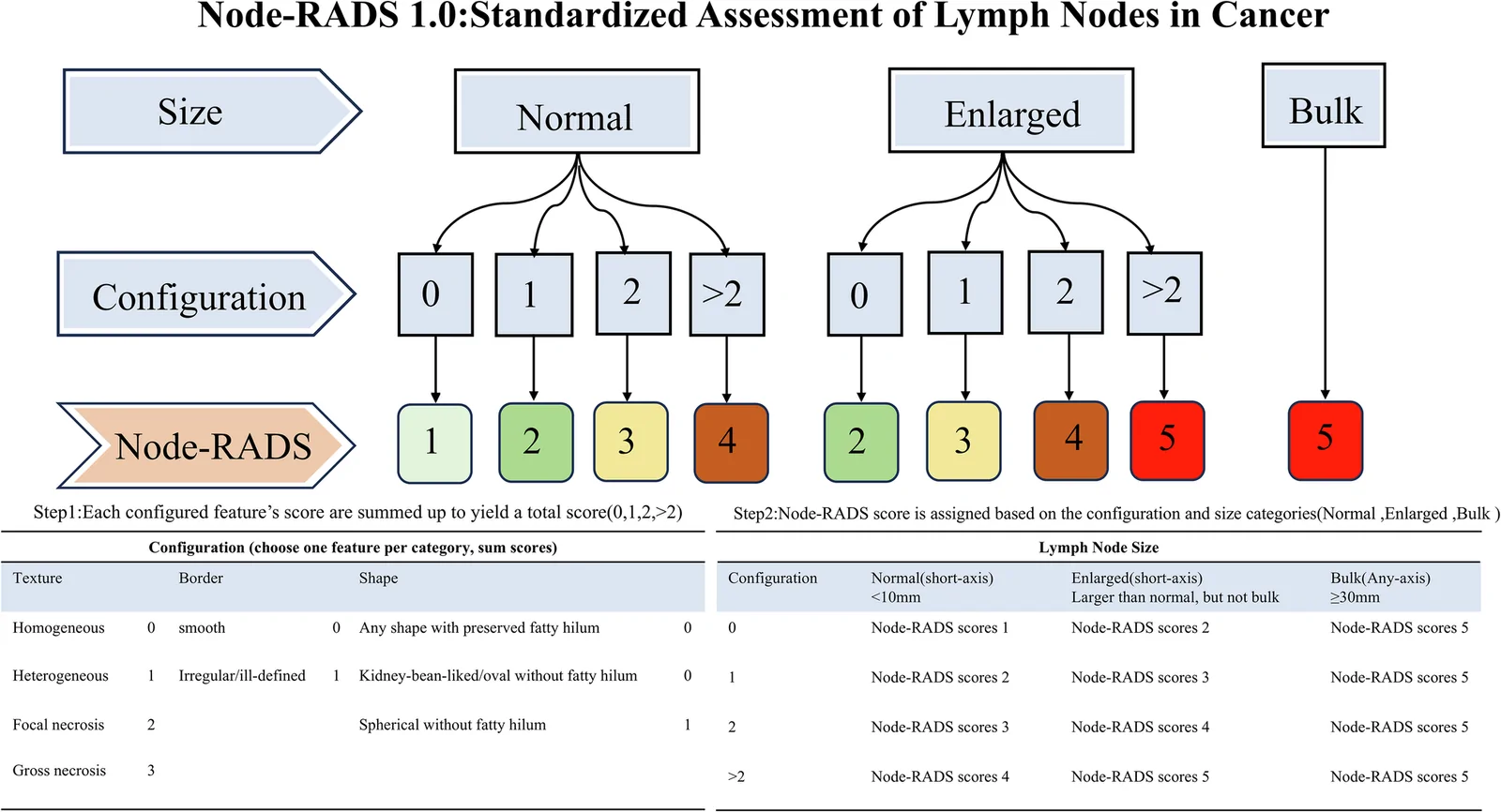
Node-RADS scoring interpretation adapted from the original source [12]

The Node-RADS scoring system (version 1.0) employs a five-point Likert scale (scores 1–5) to estimate the likelihood of LNM, with higher scores indicating a greater probability of malignancy [12]. The system integrates two principal domains: nodal size and morphological characteristics. For size evaluation, lymph nodes are categorized as normal (short-axis diameter <10 mm, 1 point), enlarged (short-axis diameter ≥ 10 mm without meeting mass criteria, or an increase of ≥ 2 mm compared with prior imaging when available, 2 points), or mass-like (maximum nodal diameter ≥ 30 mm in any dimension, 5 points). Morphological assessment comprises three components: internal texture, margin features, and shape. Internal texture and margins were scored according to predefined criteria, with texture graded from homogeneous to extensively necrotic (0–3 points) and margins classified as well-defined or irregular (0–1 point). Shape is scored as 0 points for reniform or bean-shaped nodes with a preserved fatty hilum and 1 point for round nodes lacking a fatty hilum. The final Node-RADS score is calculated as the sum of size and morphological component scores and is capped at a maximum of 5 points (Fig. 2).

### Statistical methods

**Fig. 3:**
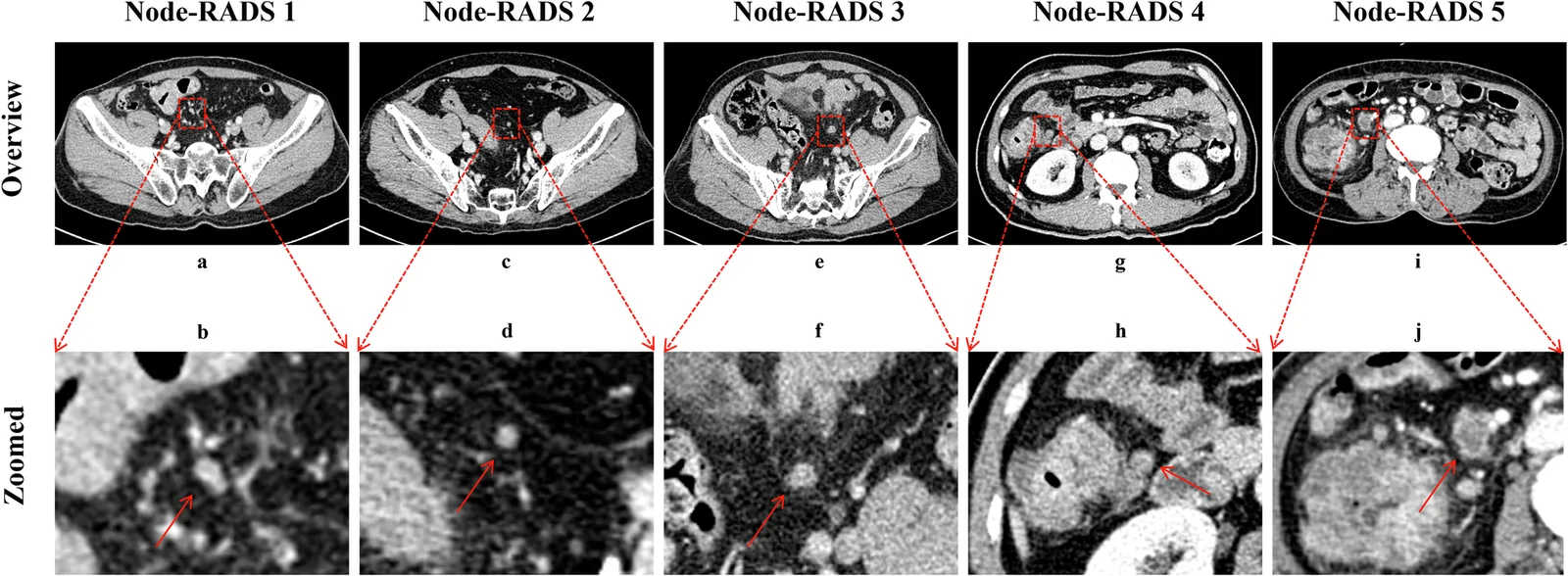
Representative examples of Node-RADS scores. Red boxes indicate the zoomed area, and arrows indicate the selected lymph node. **a**, **b** Node-RADS score: 1; short axis =  6 mm ( <10 mm), homogeneous texture (0 point), smooth border (0 point), and kidney-bean-like shape with fatty hilum (0 point). **c**, **d** Node-RADS score: 2; short axis = 6 mm ( <10 mm), homogeneous texture (0 point), smooth border (0 point), and oval shape without fatty hilum (1 point). **e**, **f** Node-RADS score: 3; short axis = 9 mm ( <10 mm), homogeneous texture (0 point), irregular border (1 point), and oval shape without fatty hilum (1 point). **g**, **h** Node-RADS score: 4; short axis = 8 mm ( <10 mm), heterogeneous texture (1 point), irregular border (1 point), and spherical shape without fatty hilum (1 point). **i**, **j** Node-RADS score: 5; short axis = 14 mm ( ≥10 mm), gross necrosis (3 points), irregular border (1 point), and spherical shape without fatty hilum (1 point)

Statistical analyses were conducted using SPSS software (version 26.0; IBM Corp). Normally distributed continuous variables are reported as mean ± standard deviation, whereas non-normally distributed variables are presented as median with interquartile range. Categorical data are expressed as counts and percentages. Diagnostic performance was evaluated using the receiver operating characteristic (ROC) curve analysis. The AUC, sensitivity, specificity, accuracy, positive predictive value (PPV), negative predictive value (NPV), and corresponding 95% confidence intervals (CIs) were calculated. Comparisons between lymph node-positive and lymph node–negative groups were performed using the Mann–Whitney *U*-test or chi-square test, as appropriate. Correlations were assessed using Spearman’s rank correlation coefficient (*r*). Differences in AUCs between models were compared using the DeLong test. Inter-observer agreement for lymph node–related imaging parameters was evaluated using ICCs and Cohen’s kappa statistics. ICC values < 0.5, 0.5–0.75, 0.75–0.9, and > 0.9 were interpreted as poor, moderate, good, and excellent agreement, respectively. Kappa values were categorized as follows: ≤ 0, no agreement; 0.01–0.20, slight; 0.21–0.40, fair; 0.41–0.60, moderate; 0.61–0.80, good; and > 0.80, excellent. A two-sided *p*-value < 0.05 was considered statistically significant (Fig. 3).

## Results

### Patient and tumor characteristics

A total of 360 patients were included in the final analysis. With regard to gender distribution, males (*n* = 200, 55.6%) slightly outnumbered females (*n* = 160, 44.4%). The age range was 27–88 years, with a median age of 63.00 (IQR: 54.00–69.00) years. In terms of tumor location, left-sided colon cancers (*n* = 225, 62.5%) were more prevalent than right-sided tumors (*n* = 135, 37.5%). Regarding histological type, adenocarcinomas (*n* = 359, 99.7%) predominated over adenosquamous carcinoma (*n* = 1, 0.3%). For tumor differentiation, the majority of cases were moderately differentiated (86.1%), whereas well-differentiated tumors were the least common (5.0%). Perineural invasion was observed in 14 patients (3.9%), and lymphovascular invasion in 20 (5.6%). In the pathological T-stage distribution, T3 was the most frequent stage (54.2%), while T1 was the least common (2.5%). All patients underwent radical lymphadenectomy. Pathological analysis identified LNM in 115 patients (31.9%), with N1 disease being more common (21.4%) than N2 (10.6%). See Table 1.

**Table 1:** Description of general information

Evaluation metrics	*n* (%)/′*x* ± s
Gender, *n* (%)	
Male	200 (55.6)
Female	160 (44.4)
Age (years), M(P_25_, P_75_)	63.00 (54.00, 69.00)
Tumor location, *n*(%)	
Right colon	135 (37.5)
Left colon	225 (62.5)
Tumor type, *n*(%)	
adenosquamous carcinoma	1 (0.3)
Adenocarcinoma	359 (99.7)
Histologic grade, *n*(%)	
Poorly differentiated	32 (8.9)
Moderately differentiated	310 (86.1)
Well differentiated	18 (5.0)
Perineural invasion, *n*(%)	14 (3.9)
Vascular invasion, *n*(%)	20 (5.6)
pT stage, *n*(%)	
1	9 (2.5)
2	26 (7.2)
3	195 (54.2)
4	130 (36.1)
pN stage, *n*(%)	
0	245 (68.1)
1	77 (21.4)
2	38 (10.6)

Inter-observer agreement across imaging parameters was good. Notably, the Node-RADS total score demonstrated excellent inter-observer reliability (ICC = 0.962), supporting its robustness and reproducibility. See Table 2.

**Table 2:** The ICC values and Kappa values of the measurements of each parameter between the two observers

Configuration	ICC/Kappa	*p*
Short axis	0.819^a^	< 0.001
Long axis	0.872^a^	< 0.001
Short-to-long axis ratio	0.765^a^	< 0.001
Heterogeneity	0.813^b^	< 0.001
Focal necrosis	0.817^b^	< 0.001
Gross necrosis	0.921^b^	< 0.001
Irregular margin	0.816^b^	< 0.001
Loss of fatty hilum	0.835^b^	< 0.001
Node-RADS	0.962^a^	< 0.001
Indistinct border	0.816^b^	< 0.001
Spiculated border	0.905^b^	< 0.001
Lobulated border	0.903^b^	< 0.001

^a^ ICC

^b^ Kappa

### Diagnostic performance of Node-RADS score and CT imaging features

Multiple imaging parameters showed significant associations with LNM. The Node-RADS total score, short-axis diameter, long-axis diameter, internal heterogeneity, focal necrosis, gross necrosis, irregular margins, loss of the fatty hilum, and spiculated or lobulated margin morphology were significantly more common in the lymph node–positive group (all *p* < 0.05). The short-to-long axis ratio and indistinct margin did not differ significantly between groups (*p* > 0.05). See Supplementary Table 1.

### Diagnostic performance of the lymph node short-axis diameter

**Fig. 4:**
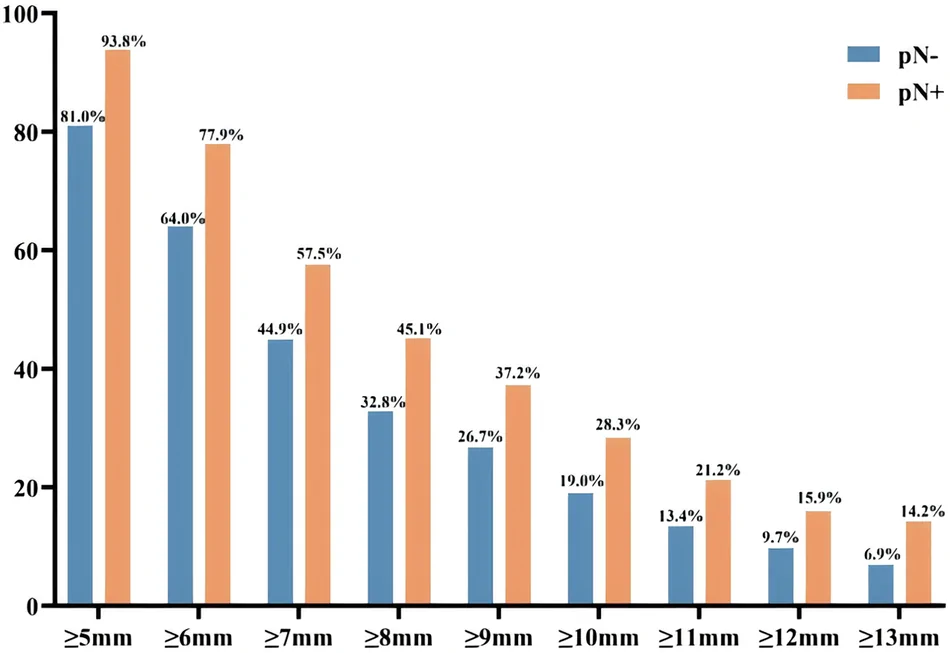
Distribution of lymph node metastasis positivity rates across different short-axis diameter cutoff values (5–13 mm) on preoperative CT

As illustrated in Fig. 4, the diagnostic performance of preoperative CT using different short-axis diameter thresholds (5–13 mm) for predicting LNM was evaluated. ROC curve analysis was conducted with histopathological findings as the reference standard across this range of cutoff values. The overall AUC was 0.570 (95% CI: 0.517–0.621), indicating limited discriminative capability. The highest diagnostic efficiency, based on the maximal Youden index, was achieved at a cutoff value of 6 mm. Detailed performance metrics—including sensitivity, specificity, accuracy, PPV, and NPV—for each threshold are presented in Table 3.

**Table 3:** ROC analysis of lymph node positivity based on different short diameter truncation values (5–13 mm) classification, %

Configuration	AUC (95% CI)	Sensitivity (95% CI)	Specificity (95% CI)	Accuracy (95% CI)	PPV (95% CI)	NPV (95% CI)
≥ 5 mm	56.4 (51.10–61.60)	93.81 (87.65–97.47)	19.03 (14.33–24.49)	42.50 (37.34–47.79)	34.64 (32.92–36.40)	87.04 (75.81–93.50)
**≥ 6 mm**	**57.0 (51.70–62.10)**	**77.88(69.10–85.14)**	**36.03 (30.04–42.36)**	**49.17 (43.89–54.46)**	**35.77 (32.72–38.95)**	**78.07 (70.81–83.94)**
≥ 7 mm	56.3 (51.00-61.50)	57.52 (47.87–66.77)	55.06 (48.63–61.37)	55.83 (50.54–61.04)	36.93 (32.19–41.95)	73.91 (68.98–78.31)
≥ 8 mm	56.2 (50.90–61.40)	45.13 (35.75–54.77)	67.21 (60.97–73.03)	60.28 (55.02–65.37)	38.64 (32.45–45.21)	72.81 (68.92–76.38)
≥9 mm	55.2 (49.90–60.40)	37.17 (28.26–46.76)	73.28 (67.30–78.69)	61.94 (56.71–66.98)	38.89 (31.68–46.62)	71.83 (68.47–74.96)
≥ 10 mm	54.6 (49.30–59.90)	28.32 (20.24–37.57)	80.97 (75.51–85.67)	64.44 (59.26–69.39)	40.51 (31.55–50.14)	71.17 (68.42–73.78)
≥ 11 mm	53.9 (48.60–59.20)	21.24 (14.11–29.93)	86.64 (81.75–90.62)	66.11 (60.97–70.99)	42.11 (31.11–53.94)	70.63 (68.35–72.81)
≥ 12 mm	53.1 (47.80–58.40)	15.93 (9.72–24.00)	90.28 (85.89–93.68)	66.94 (61.82–71.79)	42.86 (29.80–56.99)	70.13 (68.21–71.98)
≥ 13 mm	53.6 (48.30–58.90)	14.16 (8.32–21.97)	93.12 (89.21–95.94)	68.33 (63.25–73.11)	48.49 (33.05–64.22)	70.34 (68.59–72.02)

The best diagnostic performance is shown in bold, CI 95% confidence interval

### ROC analysis of diagnostic parameters for lymph node positivity

ROC curve analysis was conducted with histopathological results serving as the reference standard, and the following variables were assessed as diagnostic parameters: short-axis diameter, long-axis diameter, short-to-long axis ratio, internal heterogeneity, focal necrosis, gross necrosis, irregular margin, loss of fatty hilum, Node-RADS total score, indistinct margin, spiculated margin, and lobulated margin. The Node-RADS total score demonstrated the greatest AUC among all variables (0.835; 95% CI: 0.792–0.871; Table 4). It achieved the highest sensitivity, accuracy, and NPV (96.6%, 78.6%, and 97.8%, respectively), albeit with moderate specificity (70.5%). Among individual morphological features, focal necrosis and irregular margin showed modest discriminative ability, with AUC values of 0.606 (95% CI: 0.553–0.657) and 0.634 (95% CI: 0.582–0.684), respectively. In contrast, focal necrosis, gross necrosis, spiculated margin, and lobulated margin demonstrated high specificity (95.6%, 97.6%, 97.6%, and 92.7%, respectively) but low sensitivity (25.7%, 8.9%, 10.6%, and 17.7%, respectively), indicating that these features function primarily as rule-in indicators rather than sensitive screening markers for LNM.

**Table 4:** ROC analysis of diagnostic parameters for lymph node positivity, %

Configuration	AUC (95% CI)	Sensitivity (95% CI)	Specificity (95% CI)	Accuracy (95% CI)	PPV (95% CI)	NPV (95% CI)
Short axis	57.0 (51.70–62.10)	77.88 (69.10–85.14)	36.03 (30.04–42.36)	49.17 (43.89–54.46)	35.77 (32.72–38.95)	78.07 (70.81–83.94)
Long axis	58.50 (53.20–63.70)	81.42 (73.01–88.11)	35.63 (29.66–41.95)	50.00 (44.72–55.29)	36.65 (33.74–39.67)	80.73 (73.34–86.46)
Short-to-long axis ratio	57.20 (51.90–62.30)	77.88 (69.10–85.14)	36.44 (30.43–42.78)	49.44 (44.16–54.73)	35.92 (32.85–39.11)	78.26 (71.05–84.08)
Heterogeneity	56.80 (51.50–62.00)	45.13 (35.75–54.77)	68.42 (62.23–74.17)	61.11 (55.86–66.18)	39.54 (33.21–46.23)	73.16 (69.32–76.68)
Focal necrosis	60.60 (55.30–65.70)	25.66 (17.91–34.74)	95.55 (92.17–97.76)	73.61 (68.74–78.09)	72.50 (57.74–83.57)	73.75 (71.53–75.85)
Gross necrosis	53.20 (47.90–58.50)	8.85 (4.33–15.67)	97.57 (94.79–99.10)	69.72 (64.69–74.43)	62.50 (38.31–81.73)	70.06 (68.77–71.32)
Irregular margin	63.40 (58.20–68.40)	77.88 (69.10–85.14)	48.99 (42.60–55.40)	58.06 (52.77–63.21)	41.12 (37.38–44.96)	82.88 (77.00–87.50)
Loss of fatty hilum	58.20 (52.90–63.40)	50.44 (40.88–59.98)	65.99 (59.72–71.88)	61.11 (55.86–66.18)	40.43 (34.53–46.62)	74.43 (70.31–78.16)
**Node-RADS**	**83.50 (79.20–87.10)**	**96.46 (91.18–99.03)**	**70.45 (64.33–76.06)**	**78.61 (74.01–82.74)**	**59.89 (55.11–64.49)**	**97.75 (94.31–99.13)**
Indistinct border	55.50 (50.20–60.70)	58.41 (48.76–67.61)	52.63 (46.20–59.00)	54.44 (49.14–59.67)	36.07 (31.51–40.88)	73.45 (68.33–78.00)
Spiculated border	54.10 (48.80–59.30)	10.62 (5.61–17.82)	97.57 (94.79–99.10)	70.28 (65.26–74.95)	66.67 (43.51–83.86)	70.47 (69.07–71.83)
Lobulated border	55.20 (49.90–60.40)	17.70 (11.16–26.00)	92.71 (88.73–95.62)	69.17 (64.11–73.90)	52.63 (37.96–66.86)	71.12 (69.18–72.98)

The best diagnostic performance is shown in bold, CI 95% confidence interval

### Diagnostic performance of Node-RADS score

The AUC of the Node-RADS total score for diagnosing LNM was 0.835 (95% CI: 0.792–0.871). Based on the Youden index, an optimal diagnostic cutoff of ≥3 points was identified. As shown in Table 5, diagnostic performance metrics were evaluated across different cutoff values (≥ 2, ≥3, ≥ 4, and ≥ 5), among which a threshold of ≥3 points yielded the highest overall diagnostic efficacy. As shown in Table 4, the Node-RADS total score ( ≥3) demonstrated significantly superior diagnostic performance compared with assessment based solely on short-axis diameter. This difference was further confirmed by the DeLong test analysis, which showed that the AUC for Node-RADS ≥3 was significantly higher than that for a short-axis diameter ≥ 10 mm (0.835 vs. 0.546; *z* = 11.192, *p* < 0.001). Applying this optimal threshold, subgroup analyses revealed that the diagnostic accuracy for early-stage tumors (T1 [2.5%] and T2 [7.2%]) reached 88.9% and 92.3%, respectively, notably surpassing the overall cohort accuracy of 78.6%. Importantly, the system also maintained robust performance in complex clinical scenarios, achieving accuracies of 83.3% and 100% in patients with concomitant bowel obstruction (*n* = 48) and underlying inflammatory bowel disease (*n* = 3), respectively.

**Table 5:** ROC analysis of lymph node positivity according to different total score cutoff values (2–5 points), %

Configuration	AUC (95% CI)	Sensitivity (95% CI)	Specificity (95% CI)	Accuracy (95% CI)	PPV (95% CI)	NPV (95% CI)
Score ≥ 2	56.30 (51.00–61.50)	100.00 (96.79–100.00)	12.55 (8.69–17.34)	40.00 (34.90–45.27)	34.35 (33.29–35.42)	100.00 (88.78–100.00)
**Score** ≥**3**	**83.50 (79.20–87.10)**	**96.46 (91.18–99.03)**	**70.4 (64.33–76.06)**	**78.61 (74.01–82.74)**	**59.89 (55.11–64.49)**	**97.75 (94.31–99.13)**
Score ≥ 4	69.70 (64.70–74.40)	53.98 (44.35–63.40)	85.43 (80.40–89.58)	75.56 (70.78–79.91)	62.89 (54.51–70.56)	80.23 (76.75–83.30)
Score ≥ 5	55.10 (49.80–60.30)	15.04 (9.02–22.99)	95.14 (91.67–97.47)	70.00 (64.98–74.69)	58.62 (41.18–74.14)	71.00 (69.27–72.67)

The best diagnostic performance is shown in bold, CI 95% confidence interval

## Discussion

This study evaluated the diagnostic performance of CT-based Node-RADS for preoperative lymph node assessment in colon cancer. Our results demonstrated that Node-RADS outperformed individual imaging criteria in identifying LNM, achieving optimal diagnostic performance at a threshold of ≥3, with an AUC of 0.835 (95% CI: 0.792–0.871). Among all evaluated parameters, the Node-RADS total score provided the greatest discriminative ability by integrating multiple CT features. This advantage was further supported by the DeLong test analysis, which showed that Node-RADS performed significantly better than the conventional 10-mm size-based criterion. Importantly, inter-observer agreement for the Node-RADS total score was good, supporting the robustness and reproducibility of this structured scoring system in routine clinical practice.

The presence of LNM has a major impact on prognosis and clinical decision-making in colon cancer [3, 24]; however, consistent with previous studies, our results confirm that relying exclusively on traditional size-based criteria is insufficient [11, 25]. Although texture-related features such as internal heterogeneity have been associated with nodal metastasis in prior studies, including that by Hong et al [26], their discriminatory performance remains modest, consistent with our findings. Notably, inter-observer agreement for all imaging features in our study was good, which contrasts with the only fair overall agreement reported by Leonhardi et al [23] and may be attributed to the larger sample size of our cohort, providing more stable and reproducible feature estimates. In our cohort, focal and extensive nodal necrosis demonstrated high specificity, likely due to intratumoral necrosis from increased metabolic activity and heterogeneous vascularity [27]. However, Leonhardi et al [23] reported no significant association between texture- or necrosis-related features and nodal status, a discrepancy that may be partly attributable to differences in cohort size and statistical power. In contrast, morphological features—particularly indistinct, spiculated, and lobulated borders—were consistently associated with nodal metastasis, likely reflecting extracapsular tumor spread [28, 29]. However, these features showed high specificity but low sensitivity in our cohort, a diagnostic pattern similarly reported by Leonhardi et al [23], indicating that while configuration features are valuable rule-in signs, they are insufficient as standalone predictors of LNM.

In our study, Node-RADS ( ≥3) markedly outperformed the conventional short-axis diameter threshold of 10 mm for preoperative nodal staging in colon cancer, with a statistically significant improvement in overall discriminative ability compared with the 10-mm criterion (*p* < 0.001). Although the size-based threshold demonstrated moderate specificity, it was associated with very low sensitivity (31.3%) and a high false-negative rate (79/115), leading to substantial underestimation of LNM when size alone was used. In contrast, Node-RADS achieved excellent sensitivity (96.6%) and higher overall accuracy (78.6%), together with a markedly improved NPV, thereby substantially reducing missed metastatic lymph nodes. However, the moderate specificity (70.45%) may yield a proportion of false-positive results and overestimate nodal involvement, inadvertently leading to overtreatment, like unnecessary neoadjuvant chemotherapy. Consequently, especially for early-stage (T1/T2) patients, Node-RADS is more appropriately positioned as a rule-out tool to exclude nodal metastasis rather than a definitive rule-in diagnostic standard. Our study demonstrated that the Node-RADS scoring system provides superior diagnostic performance compared with reliance on lymph node size or isolated morphological features. Maggialetti et al [22] reported a significant association between the Node-RADS score—based on the largest detected lymph node—and pathological nodal metastasis in colon cancer, while Niu et al [30] showed that size-prioritized lymph nodes achieved better diagnostic performance, inter-reader consistency, and predictive accuracy than morphology-prioritized nodes. Collectively, these observations support that Node-RADS outperforms traditional short-axis diameter–based assessment, irrespective of whether size or morphology is emphasized. The Node-RADS score reflects an integrated evaluation of lymph node size and morphological abnormality, whereby larger nodal volume and more pronounced structural irregularities correspond to higher scores and increased malignancy risk, in agreement with prior studies and clinical observations [30, 31]. Although conditions such as bowel obstruction and inflammatory bowel disease can induce reactive changes in lymph node morphology, their confounding effects on Node-RADS performance appeared limited in our cohort, highlighting the system’s robustness in complex clinical scenarios. Nevertheless, larger targeted studies are required to fully elucidate the specific impact of these inflammatory confounders. Notably, tumor T stage remains an important clinical determinant of nodal positivity, with reported LNM rates increasing from 11.1% in T1 to 19.3% in T2 and 48.5% in T3 disease [32]. However, owing to the restricted sample size and uneven distribution of T stages in the present cohort, a comprehensive analysis of the relationship between T stage and LNM could not be performed.

Traditional free-text imaging reports frequently omit critical staging information. A survey conducted in 2018 revealed that more than half of oncologic staging reports lacked essential details required for optimal clinical decision-making [33]. In this context, analogous to established structured reporting systems such as PI-RADS [34], BI-RADS [35], and LI-RADS [36], Node-RADS can serve as a valuable adjunct to routine tumor imaging reports by promoting greater completeness, consistency, and clinical applicability. Importantly, its value lies in standardized preoperative risk stratification, particularly for nodal assessment. Emerging evidence suggests that the benefit of neoadjuvant chemotherapy is largely confined to node-positive (stage III) disease, with limited or no benefit in node-negative (stage II) patients [37]; thus, accurate preoperative N staging provided by tools such as Node-RADS may help identify patients most likely to benefit from treatment intensification.

In the Node-RADS scoring system, scores of 1–2 are generally considered negative for LNM, whereas scores of 4–5 are classified as positive. An intermediate score of 3 represents an equivocal category that should be interpreted in conjunction with primary tumor characteristics; however, the current Node-RADS framework provides no tumor-specific guidance for colorectal cancer [12]. In this study, a patient-level cutoff of ≥3 based on the highest Node-RADS score was identified as optimal, achieving an AUC of 0.835, which was superior to all alternative thresholds and individual imaging predictors (AUC range: 0.532–0.697) and consistent with prior reports [13–20]. These results support the clinical applicability of classifying Node-RADS scores < 3 as negative for LNM in colon cancer. Notably, cutoff values for Node-RADS appear to be tumor-specific; for example, in bladder cancer, a Node-RADS score < 2 has been proposed as the threshold for nodal negativity [21]. Our findings indicate substantially improved diagnostic performance of Node-RADS (AUC = 0.835) compared to earlier reports of modest efficacy [23]. This discrepancy is likely explained by our larger cohort size, which provides greater statistical power and more stable feature estimates. To our knowledge, the present study represents the largest cohort evaluating the Node-RADS system for preoperative nodal staging in colon cancer, thereby providing greater statistical stability and confidence in the observed performance. Despite the high sensitivity observed in our cohort (96.46%), specificity (70.45%) and overall accuracy (78.61%) remained moderate. By comparison, studies in head and neck squamous cell carcinoma reported lower sensitivity (61.54%) but higher specificity (89.69%) and accuracy (87.29%) [13], while investigations in esophageal squamous cell carcinoma demonstrated both high sensitivity (96.5%) and specificity (92.0%) [16]. The relatively lower specificity and accuracy observed in our study likely reflect intrinsic differences among tumor types and patient populations, including variations in stage distribution, metastatic patterns, and underlying tumor biology.

This study has several limitations. First, the single-center retrospective design, with inclusion restricted to patients undergoing upfront surgery and an imbalanced distribution of T stages within the cohort, may have introduced selection bias. This could, to some extent, limit the generalizability of our findings to non-surgical populations. Therefore, validation in larger, multicenter prospective studies is warranted. Second, analyses were conducted at the patient level rather than at the individual lymph node level, which precluded direct node-by-node correlation between imaging findings and histopathological results. In addition, metastatic lymph nodes and tumoral deposits were not specifically distinguished on imaging, which may have introduced potential bias in nodal assessment. Future studies should apply Node-RADS to each detectable lymph node on CT and validate imaging assessments against surgical specimens. Third, lymph nodes smaller than 5 mm were excluded because of limited delineation on CT, potentially leading to underestimation of micrometastases. Finally, quantitative imaging features such as radiomics were not incorporated; these objective measures may further improve diagnostic performance and could be integrated into future iterations of Node-RADS.

## Conclusion

In summary, Node-RADS provides reliable diagnostic performance for the assessment of LNM in colon cancer and offers meaningful improvement over traditional diagnostic approaches. Its structured and reproducible framework facilitates standardized reporting and improves the clarity and clinical utility of radiologic assessments.

## Supplementary information

Electronic supplementary material

## Data Availability

All data are included in this article and its supplementary materials.

## Abbreviations

AUC: Area under the curve
CI: Confidence interval
CT: Computed tomography
ICC: Intraclass correlation coefficient
LNM: Lymph node metastasis
Node-RADS: Node-Reporting and Data System
NPV: Negative predictive value
PPV: Positive predictive value
ROC: Receiver operating characteristic

## References

[1] Siegel RL, Wagle NS, Cercek A et al (2023) Colorectal cancer statistics, 2023. CA Cancer J Clin 73:233–254 10.3322/caac.21772 36856579

[2] Sung H, Ferlay J, Siegel RL et al (2021) Global Cancer Statistics 2020: GLOBOCAN estimates of incidence and mortality worldwide for 36 cancers in 185 countries. CA Cancer J Clin 71:209–249 10.3322/caac.21660 33538338

[3] Taylor FGM, Quirke P, Heald RJ et al (2011) Preoperative high-resolution magnetic resonance imaging can identify good prognosis stage I, II, and III rectal cancer best managed by surgery alone: a prospective, multicenter, European study. Ann Surg 253:711–719 10.1097/SLA.0b013e31820b8d52 21475011

[4] Haddad P, Ghalehtaki R, Saeedian A et al (2021) Current approaches in intensification of long-course chemoradiotherapy in locally advanced rectal cancer: a review. Radiat Oncol J 39:83–90 10.3857/roj.2021.00108 PMC8497854 34619824

[5] Marijnen CAM, Van De Velde CJH, Putter H et al (2005) Impact of short-term preoperative radiotherapy on health-related quality of life and sexual functioning in primary rectal cancer: report of a multicenter randomized trial. J Clin Oncol 23:1847–1858 10.1200/JCO.2005.05.256 15774778

[6] Huang Q, Wu X, Wang Z et al (2022) The primordial differentiation of tumor-specific memory CD8+ T cells as bona fide responders to PD-1/PD-L1 blockade in draining lymph nodes. Cell 185:4049–4066.e25 10.1016/j.cell.2022.09.020 36208623

[7] Du Bois H, Heim TA, Lund AW (2021) Tumor-draining lymph nodes: At the crossroads of metastasis and immunity. Sci Immunol 6:eabg3551 10.1126/sciimmunol.abg3551 PMC8628268 34516744

[8] Sikkenk DJ, Henskens IJ, Van De Laar B et al (2024) Diagnostic performance of MRI and FDG PET/CT for preoperative locoregional staging of colon cancer: systematic review and meta-analysis. AJR Am J Roentgenol 223:e2431440 10.2214/AJR.24.31440 39230407

[9] Benson AB, Hunt S, Parikh A (2024) Colon Cancer, Version 3.2024, NCCN Clinical Practice Guidelines in Oncology. J Natl Compr Canc Netw 22:e240029 10.6004/jnccn.2024.0029 38862008

[10] Miao S, Lu Y, Chen H et al (2020) Contrast-enhanced CT imaging for the assessment of lymph node status in patients with colorectal cancer. Oncol Lett 19:3451–3458 10.3892/ol.2020.11454 PMC7114938 32269618

[11] Filippone A, Ambrosini R, Fuschi M et al (2004) Preoperative T and N staging of colorectal cancer: accuracy of contrast-enhanced multi–detector row CT colonography—initial experience. Radiology 231:83–90 10.1148/radiol.2311021152 14990815

[12] Elsholtz FHJ, Asbach P, Haas M et al (2021) Introducing the Node Reporting and Data System 1.0 (Node-RADS): a concept for standardized assessment of lymph nodes in cancer. Eur Radiol 31:6116–6124 10.1007/s00330-020-07572-4 PMC8270876 33585994

[13] Lorusso G, Villanova I, Greco S et al (2025) Accuracy of MRI-based Node-RADS in predicting metastatic lymph node involvement in Head and Neck Squamous Cell Carcinoma. Eur J Radiol 186:112051 10.1016/j.ejrad.2025.112051 40117957

[14] Meyer HJ, Schnarkowski B, Pappisch J et al (2022) CT texture analysis and node-RADS CT score of mediastinal lymph nodes—diagnostic performance in lung cancer patients. Cancer Imaging 22:75 10.1186/s40644-022-00506-x PMC9791752 36567339

[15] Pediconi F, Maroncelli R, Pasculli M et al (2024) Performance of MRI for standardized lymph nodes assessment in breast cancer: are we ready for Node-RADS? Eur Radiol 34:7734–7745 10.1007/s00330-024-10828-y PMC11557668 38867119

[16] Fang Y, Chen M, Zheng X et al (2024) Validation of the Node Reporting and Data System (Node-RADS) for standardized CT evaluation of regional lymph nodes in esophageal squamous cell carcinoma patients. Eur Radiol 35:2999–3009 10.1007/s00330-024-11234-0 39613956

[17] Sun Y, Xiao H, Wen L et al (2025) Value of post-NAC CT-based Node-RADS score for Predicting residual lymph node metastasis and survival outcome of locally advanced gastric cancer. Abdom Radiol (NY) 50:3936–3949 10.1007/s00261-025-04843-w 39948223

[18] Li X, Lin D, Xiong Y et al (2025) Node-RADS category on preoperative CT predicts prognosis in patients with papillary renal cell carcinoma. Eur Radiol 35:5480–5491 10.1007/s00330-025-11446-y PMC12350559 39979621

[19] Wu Q, Lou J, Liu J et al (2024) Performance of node reporting and data system (node-RADS): a preliminary study in cervical cancer. BMC Med Imaging 24:28 10.1186/s12880-024-01205-8 PMC10811875 38279127

[20] Jiang L, Zhuang Z, Tang X et al (2025) Diagnostic performance of node-RADS classification for primary lymph node assessment in rectal cancer: a modality benchmarking study. J Cancer Res Clin Oncol 151:145 10.1007/s00432-025-06196-9 PMC12009234 40252124

[21] Leonardo C, Flammia RS, Lucciola S et al (2023) Performance of Node-RADS Scoring System for a standardized assessment of regional lymph nodes in bladder cancer patients. Cancers (Basel) 15:580 10.3390/cancers15030580 PMC9913205 36765540

[22] Maggialetti N, Greco CN, Lucarelli NM et al (2023) Applications of new radiological scores: the Node-RADS in colon cancer staging. Radiol Med 128:1287–1295 10.1007/s11547-023-01703-9 37704777

[23] Leonhardi J, Mehdorn M, Stelzner S et al (2024) Diagnostic accuracy and reliability of CT-based Node-RADS for colon cancer. Abdom Radiol 50:1–7 10.1007/s00261-024-04485-4 PMC11711253 38976057

[24] Argilés G, Tabernero J, Labianca R et al (2020) Localised colon cancer: ESMO Clinical Practice Guidelines for diagnosis, treatment and follow-up. Ann Oncol 31:1291–1305 10.1016/j.annonc.2020.06.022 32702383

[25] Shkurti J, Van Den Berg K, Van Erning FN et al (2023) Diagnostic accuracy of CT for local staging of colon cancer: a nationwide study in the Netherlands. Eur J Cancer 193:113314 10.1016/j.ejca.2023.113314 37729742

[26] Hong EK, Landolfi F, Castagnoli F et al (2021) CT for lymph node staging of Colon cancer: not only size but also location and number of lymph node count. Abdom Radiol (NY) 46:4096–4105 10.1007/s00261-021-03057-0 33904991

[27] Li S, Li Z, Wang L et al (2023) CT morphological features for predicting the risk of lymph node metastasis in T1 colorectal cancer. Eur Radiol 33:6861–6871 10.1007/s00330-023-09688-9 37171490

[28] D’journo XB, Avaro JP, Michelet P et al (2009) Extracapsular lymph node involvement is a negative prognostic factor after neoadjuvant chemoradiotherapy in locally advanced esophageal cancer. J Thorac Oncol 4:534–539 10.1097/jto.0b013e31819c862d 19347985

[29] Liu D, Lan Y, Zhang L et al (2021) Nomograms for predicting axillary lymph node status reconciled with preoperative breast ultrasound images. Front Oncol 11:567648 10.3389/fonc.2021.567648 PMC8058421 33898303

[30] Niu Y, Wen L, Yang Y et al (2024) Diagnostic performance of Node Reporting and Data System (Node-RADS) for assessing mesorectal lymph node in rectal cancer by CT. BMC Cancer 24:716 10.1186/s12885-024-12487-0 PMC11165899 38862951

[31] Matsuoka H, Nakamura A, Masaki T et al (2007) Optimal diagnostic criteria for lateral pelvic lymph node metastasis in rectal carcinoma. Anticancer Res 27:3529–3533 17972513

[32] Wu Z, Qin G, Zhao N et al (2017) Assessing the adequacy of lymph node yield for different tumor stages of colon cancer by nodal staging scores. BMC Cancer 17:498 10.1186/s12885-017-3491-2 PMC5526283 28743236

[33] Patel A, Rockall A, Guthrie A et al (2018) Can the completeness of radiological cancer staging reports be improved using proforma reporting? A prospective multicentre non-blinded interventional study across 21 centres in the UK. BMJ Open 8:e018499 10.1136/bmjopen-2017-018499 PMC6169672 30282676

[34] Weinreb JC, Barentsz JO, Choyke PL et al (2016) PI-RADS Prostate Imaging–Reporting and Data System: Version 2. Eur Urol 69:16–40 10.1016/j.eururo.2015.08.052 PMC6467207 26427566

[35] Lazarus E, Mainiero MB, Schepps B et al (2006) BI-RADS lexicon for US and mammography: interobserver variability and positive predictive value. Radiology 239:385–391 10.1148/radiol.2392042127 16569780

[36] Chernyak V, Fowler KJ, Do RKG et al (2023) LI-RADS: looking back, looking forward. Radiology 307:e222801 10.1148/radiol.222801 PMC10068888 36853182

[37] Horesh N, Emile SH, Freund MR et al (2025) Neoadjuvant chemotherapy improves overall survival in stage III but not in stage II colon cancer: a propensity score-matched analysis of the National Cancer Database. Surgery 183:109389 10.1016/j.surg.2025.109389 40344993

