# Supplementary material for: Clinical validation of Node-RADS for nodal staging in colon cancer: a retrospective study of 360 patients
Source: Insights Imaging. 2026 Nov 2;17:275. doi: 10.1186/s13244-026-02441-y (PMC13728009; doi:10.1186/s13244-026-02441-y)
Supplement: Supplementary file 1 [file 13244_2026_2441_MOESM1_ESM.pdf]

Clinical Validation of Node-RADS for Nodal Staging in Colon Cancer: A Retrospective Study of 360 Patients

ELECTRONIC SUPPLEMENTARY MATERIAL

Supplementary Table S1 Comparison of CT Imaging Features and Node-RADS Scores Between Lymph Node–Positive and Lymph Node–Negative Groups

Configuration	Lymph Node Metastasis		
	χ^2/z	*P*	*r*
Short axis	-3.128[a]	0.002	0.165
Long axis	-2.744[a]	0.006	0.145
Short-to-long axis ratio	-0.578[a]	0.563	0.031
Heterogeneity	6.194[b]	0.013	0.131
Focal necrosis	35.315[b]	<0.001	0.313
Gross necrosis	7.525[b]	0.006	0.145
Irregular margin	23.209[b]	<0.001	0.254
Loss of fatty hilum	8.789[b]	0.003	0.156
Node-RADS	-10.981[a]	<0.001	0.580
Indistinct border	3.780[b]	0.052	0.102
Spiculated border	10.949[b]	0.001	0.174
Lobulated border	8.902[b]	0.003	0.157

a:Mann-Whitney U test, b: the chi-square test, r: the correlation coefficient.
